# The “Median” Method for the Reduction of Noise and Trigger Jitter on Waveform Data

**DOI:** 10.6028/jres.110.079

**Published:** 2005-10-01

**Authors:** N. G. Paulter, D. R. Larson

**Affiliations:** National Institute of Standards and Technology, Gaithersburg, MD 20899-0001

**Keywords:** aberration, median, median method, noise, overshoot, transition duration, trigger jitter, waveform

## Abstract

The “median” method for the reduction of the effect of noise and trigger jitter on waveform data is described. The effectiveness of this method was examined using simulations and experiments and, for typical jitter and noise observed in electrical pulse metrology, is shown to provide reconstructed waveforms with transition durations that accurately match those of the input signal. Also, for aberrations, an upper bound on the error in the amplitude of the aberration is provided.

## 1. Introduction

The effect of trigger jitter on the measurement of signals is to add signal-dependent noise and to reduce the measurement bandwidth or, equivalently, to slow the step response of the measurement instrumentation. This reduction or slowing results in an increase in the transition duration, *t*_sys_, of the step response of the measurement system. *t*_sys_ can be approximated using:

$$t_{\text{sys}}=\sqrt{t_{\text{instr}}^{2}+t_{\text{jit}}^{2}},$$ (1)

where *t*_instr_ is the transition duration of the step response of the measurement instrument and *t*_jit_ is the transition duration of the cumulative jitter distribution. The relationship between *t*_jit_ and the measured value of the rms jitter, *σ*_jit_, can be found either by calculation and tables [1] or by waveform simulation and extraction of waveform parameters, both of which yield *t*_jit_ = 2.56 *σ*_jit_.

If *t*_jit_ < 0.1 *t*_instr_, then in most cases *t*_jit_ can be ignored. However, in high-speed pulse metrology, *t*_jit_ is often a large fraction of *t*_instr_. For example, in some samplers with 3 dB attenuation bandwidths of 80 GHz, *t*_jit_ ≈ 1 ps and *t*_instr_ ≈ 5 ps. Jitter in this case must be removed to get an accurate estimate of pulse parameters and an accurate reconstruction of the input signal. Since jitter effectively acts as a lowpass filter [2], it can be removed via deconvolution. Although uncertainties associated with the deconvolution process can be assigned to jitter deconvolution [3], it would be advantageous to pulse metrology if an alternative method with lower uncertainties could be developed. Furthermore, the presence of noise increases the uncertainties associated with the deconvolution or waveform reconstruction process [4,5]. Also, typical waveform reconstruction methods employ a regularization operator that comprises a second order difference operator. This difference operator typically causes ringing near waveform transitions [5] and, thereby, may increase the amplitudes of overshoot, undershoot, and other waveform aberrations. Noise in the measurement is reduced by signal averaging; this reduction is proportional to *M*^−1/2^, where *M* is the number of waveforms averaged.

Each of the *m* (*m* = 1, 2, …, *M*) waveforms, *f_m_*[*t_n_*] (*n* = 1, 2, …, *N*, where *N* is the number of samples in a waveform) is described to first order by:

$${f_{m}\left[t_{n}\right]=f_{0}\left[t_{n}\right]+\frac{\partial f_{0}}{\partial t})}_{t_{n}}\times j_{m}\left[t_{n}\right]+s_{m}\;\left[t_{n}\right],$$ (2)

where *f*_0_ is the jitter-free, noise-free waveform, *j_m_* is the trigger jitter corresponding to the *m*th waveform, *s_m_* is the noise corresponding to the *m*th waveform, *t_n_* is discrete time with index *n*, and $\frac{\partial f_{0}}{\partial t}$ is the derivative of the signal in the vicinity of *t_n_*. The average of Eq. (2), 〈*f* [*t_n_*]*_M_*〉, is the value typically provided by oscilloscopes or other waveform recorders:

$${\langle f\left[t_{n}\right]\rangle }_{M}=f_{0}\left[t_{n}\right]+g_{M}\;\left[t_{n}\right]+{\langle s\left[t_{n}\right]\rangle }_{M},$$ (3)

where 〈*s*[*t_n_*]*_M_*〉 is the mean value of the noise at *t_n_* and *g_M_*[*t_n_*] is the amplitude contribution from jitter at *t_n_*. Based on our observations and the physical cause of the distributions of *s* and *j*, both distributions are normal with zero mean and are independent and identically distributed for each *t_n_* and each waveform. The two distributions, one caused by jitter and the other by noise, can be dealt with independently. For noise, therefore, we can write:

$${\langle s\left[t_{n}\right]\rangle }_{M}=\langle s\left[t_{n}\right]\rangle =\langle s\rangle .$$ (4)

For noise and for large *M*, the median, mode, and mean of 〈*s*[*t_n_*]*_M_*〉 will yield the same value for a symmetric unimodal distribution, which is 〈*s*〉, where 〈*s*〉 = 0 for the situation described herein. This is not the case for the effect of jitter because the coefficient of *j* is signal (data) dependent [(see Eq. (2)]. In the case where the mean estimator is used to approximate the signal, jitter will act as a low pass filter [2] thereby reducing the bandwidth of the signal and increasing its transition duration. The median and mode do not involve averaging, and they do not bandlimit the signal as strongly as does the mean. However, as will be shown later, the ability to accurately reconstruct waveform aberrations using the median is limited by the duration of the aberrations relative to *σ*_jit_. The use of the word aberration and how to compute its values are defined in Ref. [6].

The method we have examined to reduce the effects of noise and trigger jitter on the reconstructed waveform is based on taking the median value for each instant, *t_n_*, of the set of *M* waveforms. Therefore, instead of letting the oscilloscope average a set of *M* waveforms to yield one waveform that is typically displayed on the oscilloscope, a set of *M* unaveraged waveforms are acquired by a computer to provide a two-dimensional array of *M* unaveraged waveforms each with *N* samples. At each *t_n_* the median is computed, and the results recorded to yield the reconstructed waveform. We also examined using the mode to remove jitter, but the results for the pulse parameter values showed greater variation than that from using the median. Also the waveform root-sum-of-squares error was greater for the mode than for the median. Although we have implemented the median method on a computer after acquisition of all *M* waveforms, it could also be implemented in the firmware of the oscilloscope prior to acquisition of the resultant (median reconstructed) waveform, just as signal averaging is presently implemented.

The median method has also been examined by other researchers [7,8]. As they [7,8] both point out, the median method works well for monotonic signals but introduces errors when the signal is not monotonic (that is, exhibits a local extremum). The duration of this “monotonicity,” however, need only be long compared to *σ*_jit_ (this is discussed in Sec. 5). The median method causes the magnitudes of the local extrema to be underestimated. In Sec. 5, we provide an upper bound for this error and show that it is small compared to the amplitude of the aberration when the duration of the aberration is small compared to *σ*_jit_. In [8], the author opts for a jitter deconvolution method rather than the median to improve the accuracy of the reconstructed spectrum (the higher frequency components are increased). However, the nature of the regularization operator in waveform reconstructions using deconvolution will cause a false increase in the magnitude of the high-frequency components simply because of the spurious ringing introduced near transitions (see Sec. 5). The magnitude of this ringing will depend on, for example, the accuracy of the estimate of the jitter function and signal noise. An algorithm for removing noise and jitter from waveforms using spline approximations was also examined [9]. No pulse parameter values were provided in [9], consequently, a quantitative comparison was not possible. However, by comparing figures of waveforms generated with similar conditions of noise and jitter relative to pulse amplitude and transition duration (or pulse duration), it appears the waveforms extracted using the median method exhibit less departure from the target values in the vicinities of aberrations than does the spline method.

## 2. Simulation Results of Ideal Step Waveforms

**Fig. 1:**
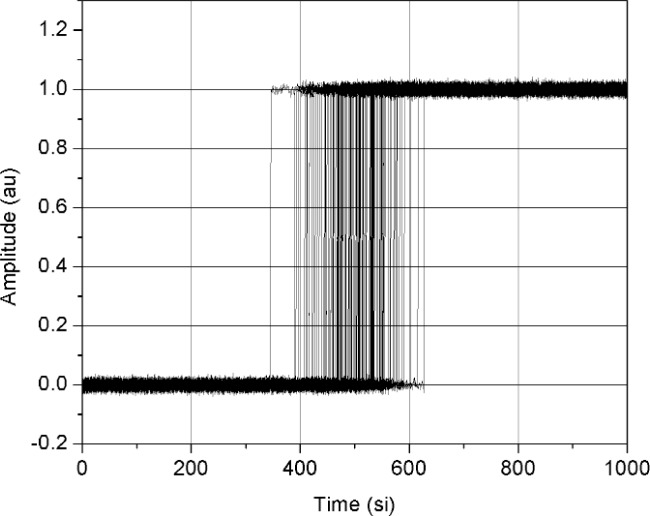
Set of 128 jittered ideal steps with additive noise. Noise level is 0.01 au rms and jitter is 50 si rms. (au = amplitude unit, si = sampling interval).

**Fig. 2:**
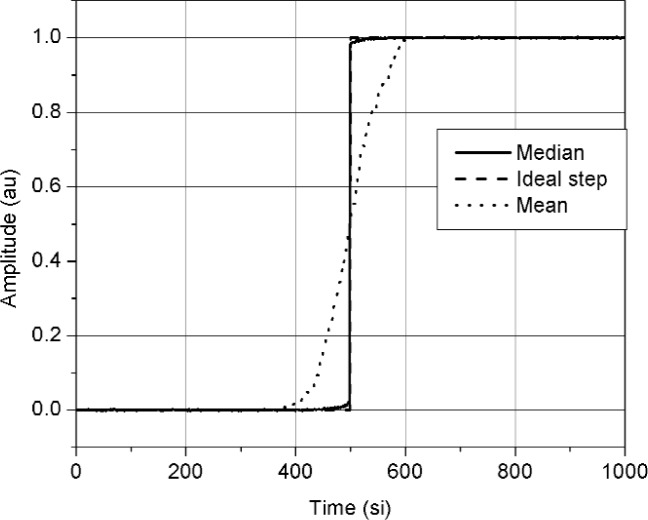
The median reconstructed step (solid line), the ideal jitter-free, noise-free step (dashed line), and the mean of the 128 steps shown in Fig. 1 (dotted line). (au = amplitude unit, si = sampling interval).

**Fig. 3:**
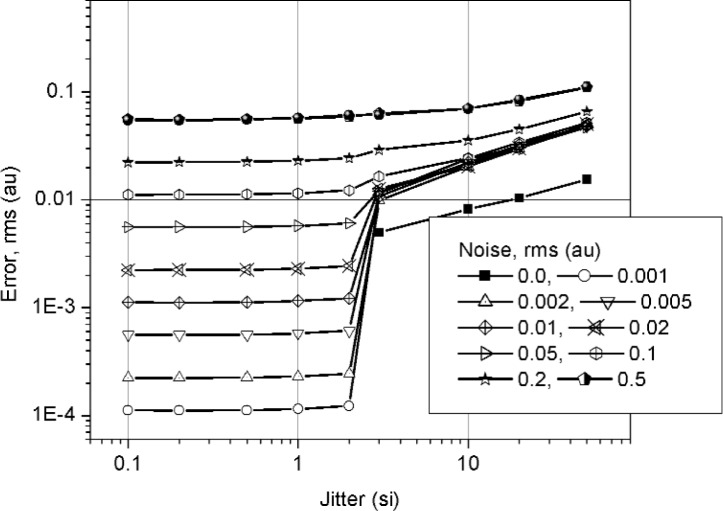
Rms error in median reconstructed waveforms. Each datum is the result of 100 averages. (au = amplitude unit, si = sampling interval).

We examined the median method for jitter and noise reduction by using simulations and experiments. In the first set of simulations, we used a jittered ideal step with additive noise and examined the error in the reconstructed waveforms as a function of noise and jitter. The additive noise and trigger jitter were both modeled as having a zero-mean normal distribution. The amplitude, *A_p_*, of the step was 1 amplitude unit (au); the rms additive noise, *σ_n_*, as a percentage of *A_p_*, ranged between 0 % and 50 % (or, 0 au to 0.5 au); and the rms jitter, in units of a sampling interval (si), ranged from 0 si to 50 si. Figure 1 shows an example of one set of 128 jittered noisy waveforms (for the set shown, *σ*_jit_ = 50 si, *σ_n_* = 0.01 *A_p_*). Figure 2 shows the waveform reconstructed using the median method, the input (ideal step) waveform, and the waveform that is the result of the average of the waveforms shown in Fig. 1. As can be seen from Fig. 2, the reconstructed step closely resembles the input step even for large *σ*_jit_. Figure 3 shows the rms error value of the median reconstructed steps as a function of noise and trigger jitter. The rms error is given by:

$$e={\left\{\frac{1}{K}\sum_{k=1}^{K}\sqrt{\frac{1}{N}\sum_{n=1}^{N}{\left(s_{n,k}-s_{n,\text{ideal}}\right)}^{2}}\right\}}^{1/2},$$ (5)

where *K* is the number of iterations (100 in this case), *s_n_* is the value of the step at index *n*, *s_n_*_,ideal_ is the value of ideal step at index *n*, and *N* is the number of samples (1000 in this case). Since the ideal step had a 50 % duty factor and a low and high state value of 0 au and 1 au, its rms value is 0.707 au. Comparing this value to the rms error shown in Fig. 3, it can be seen that the median reconstruction method significantly reduces noise and jitter with respect to the input waveforms.

## 3. Simulation Results of Step-like Waveforms

In the second set of simulations, we used the same ideal step but filtered it with a Butterworth filter (order = 3, low-frequency-pass cut off = 0.125/si, and sampling frequency = 2/si) to give the signal features (such as aberrations) typically observed in pulse waveforms. The pulse parameters of transition duration (between the instants corresponding to 10 % and 90 % of pulse amplitude), *t*_10–90_, and overshoot, *OS*, were compared. The process of determining the pulse parameters starts with obtaining the bimodal histogram of the waveform amplitude values, per methods described in [6]. The methods to compute transition duration and overshoot are given in [6].

**Fig. 4:**
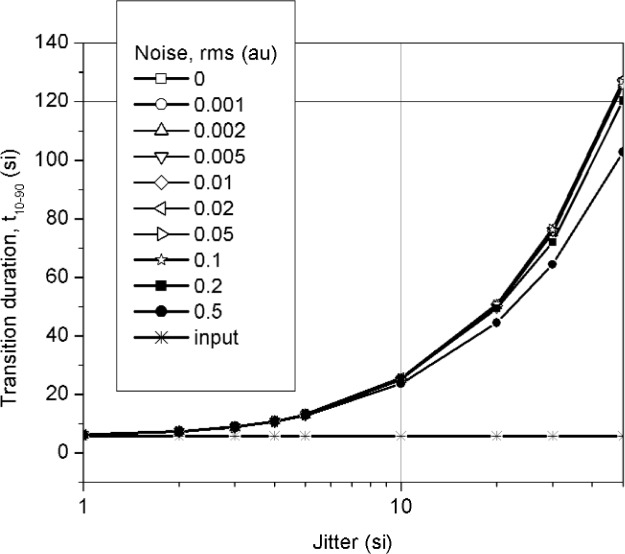
Transition duration (*t*_10–90_) for the waveform obtained from the computer average of 128 unaveraged waveforms. Each datum represents the average of 100 iterations. The line labeled “input” represents the target value. (au = amplitude unit, si = sampling interval).

**Fig. 5:**
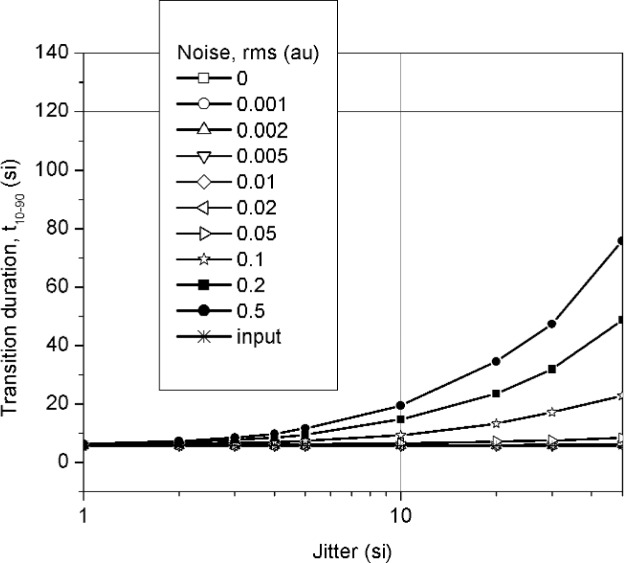
Transition duration (*t*_10–90_) for the waveform based on the median of 128 unaveraged waveforms. Each datum represents the average of 100 iterations. The line labeled “input” represents the target value. (au = amplitude unit, si = sampling interval).

**Fig. 6:**
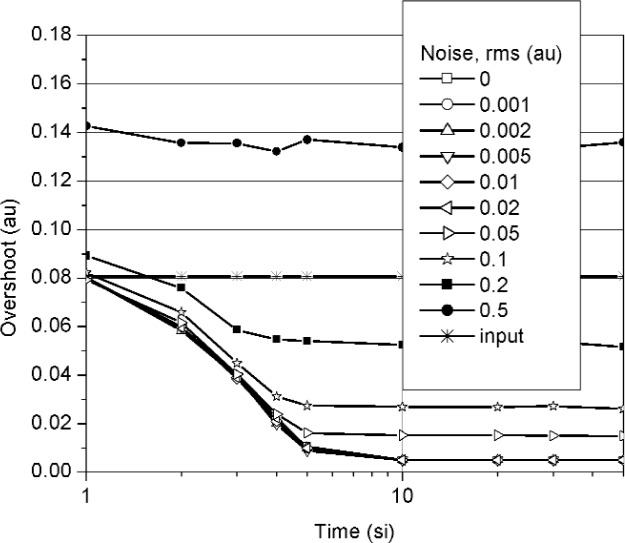
Overshoot for the waveform obtained from the computer average of 128 unaveraged waveforms. Each datum represents the average of 100 iterations. The line labeled “input” represents the target value. (au = amplitude unit, si = sampling interval).

**Fig. 7:**
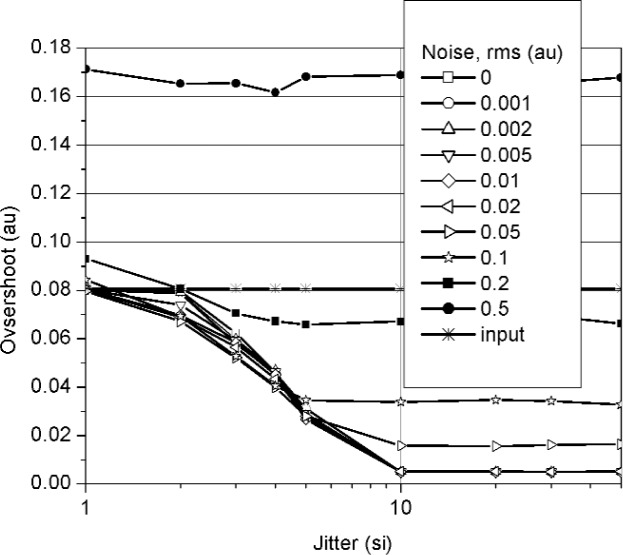
Overshoot for the waveform based on the median of 128 unaveraged waveforms. Each datum represents the average of 100 iterations. The line labeled “input” represents the target value. (au = amplitude unit, si = sampling interval).

**Fig. 8:**
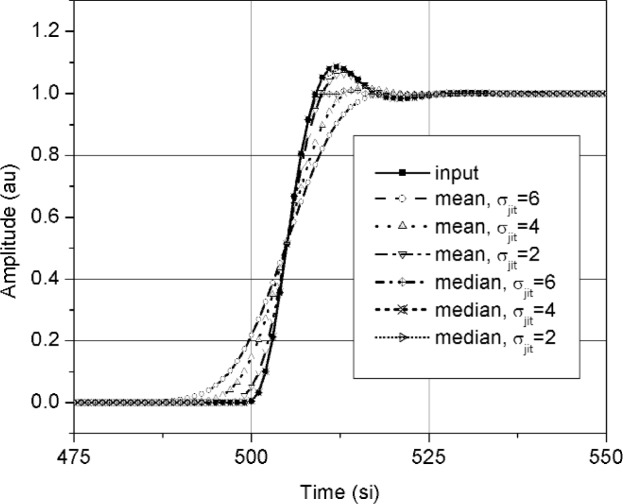
Butterworth filtered noise-free step-like waveforms with different levels of jitter for both the median reconstructed waveform and the averaged waveform. Also shown is the input waveform. (au = amplitude unit, si = sampling interval).

The noise-free waveform had a *t*_10–90_ = 5.86 si and an *OS* of 0.08 au. Overshoot was examined because it is the aberration with the largest amplitude and would be the most obviously sensitive to jitter. The duration of the overshoot (between its 50 % reference level instants, as defined in Ref. [6]) was approximately 1 si. Figures 4 and 5 show the effect of trigger jitter and noise on the transition duration of the averaged and median reconstructed waveforms. As can be seen, the transition duration of the averaged waveform is very sensitive to jitter and noise. The median-reconstructed waveform provides waveforms that accurately reproduce the transition duration of the input signal for *σ*_jit_ ≤ 10 si and *σ_n_* ≤ 0.1 au. Figures 6 and 7 show the effect of trigger jitter and noise on the overshoot of the averaged and reconstructed waveforms. As can be seen, the overshoot of the averaged waveform is very sensitive to jitter and noise. The overshoot in the median reconstructed waveform is also sensitive to jitter and noise. The large error in overshoot for large jitter (*σ*_jit_ > 1 si) is caused by the fact that both the positive and negative transitions of the overshoot contribute to the set of data values in the vicinity of the overshoot. This effect is more clearly shown in Fig. 8 where different median-reconstructed waveforms are shown along with the averaged waveforms. In this figure, the duration, *T_OS_*, (between 50 % reference levels) of the overshoot is approximately 5 si and the transition duration of the cumulative jitter distribution is 2.56 *σ*_jit_. The results of Fig. 8 indicate that integrity of the reconstructed waveform is better for *σ*_jit_ < *T_OS_*. *OS* in the median reconstructed waveform is not sensitive to noise for *σ_n_* ≤ 0.1 au. The primary purpose for showing Figs. 4 and 6 is as a reference for the effects of averaging on overshoot and transition duration in the presence of noise and trigger jitter.

**Fig. 9:**
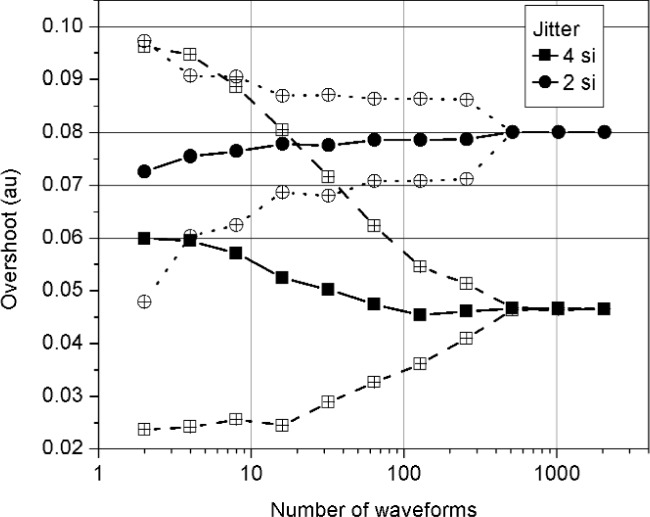
The overshoot as a function of the number, *M*, of waveforms used in the median reconstruction method. Each datum represents the average of 100 iterations of a simulation using *M* waveforms. The hollow circles and squares represent the 2-sigma uncertainty bounds. The target value for overshoot is 0.08 au. (au = amplitude unit, si = sampling interval).

**Fig. 10:**
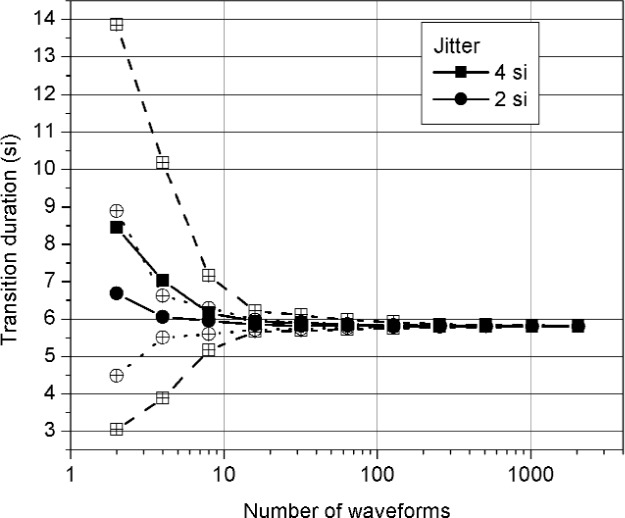
The transition duration as a function of the number, *M*, of waveforms used in the median reconstruction method. Each datum represents the average of 100 iterations of a simulation using *M* waveforms. The hollow circles and squares represent the 2-sigma uncertainty bounds. The target value for transition duration is 5.86 si. (au = amplitude unit, si = sampling interval).

Figures 9 and 10 show the effect of the number of waveforms, *M*, used in the simulation to compute the median for *σ*_jit_ = 2 si and 4 si. The generated waveforms were noise free. Each datum in Figs. 9 and 10 is the result of repeating the simulation 100 times for each set of *M* waveforms. The transition duration values show a monotonic decrease in value, for both levels of jitter, to the target value of 5.86 si. The overshoot values, on the other hand, do not exhibit a common monotonic change approaching the target value of 0.08 au. For *σ*_jit_ ≥ 4 si, increasing *M* does not result in the correct value of 0.08 au for overshoot. As discussed earlier, this is because the duration of the overshoot is less than the transition duration of the cumulative jitter distribution. For *σ*_jit_ = 2 si, on the other hand, the target value of 0.08 au for overshoot is obtained. Figures 9 and 10 also show that *M* ≥ 512 is required to achieve convergence to the correct value. Furthermore, the variation in the values of overshoot and transition duration are significant for *M* < 128.

## 4. Experimental Results

**Fig. 11:**
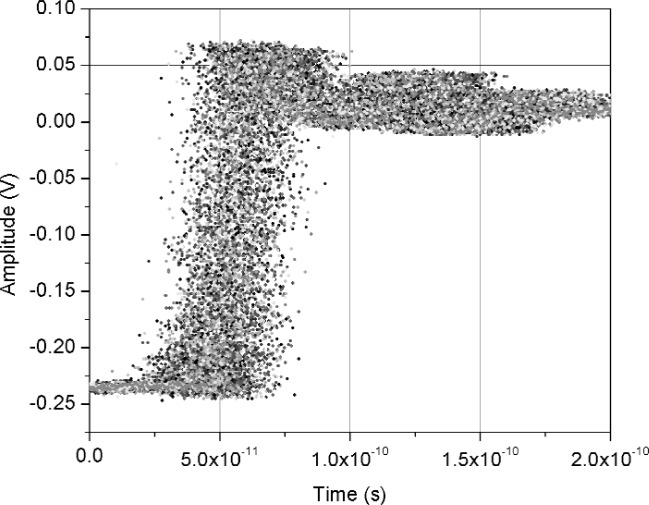
Set of jittered noisy waveforms acquired from sampler A, where the input jitter value was measured to be 9.48 ps rms.

**Fig. 12:**
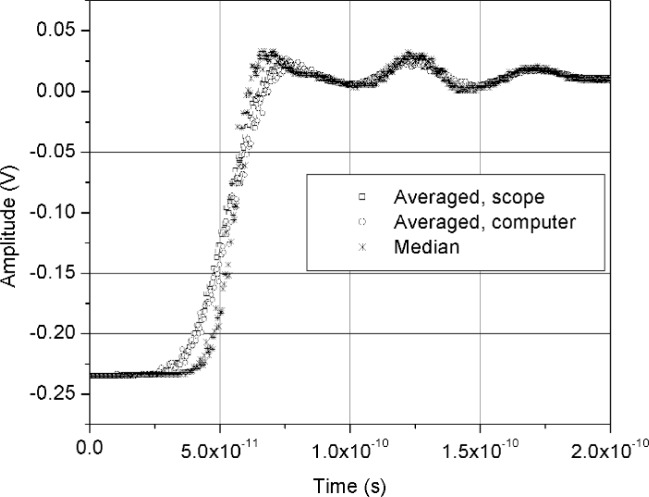
The scope averaged, computer averaged, and median reconstructed waveforms corresponding to the data set shown in Fig. 11.

**Fig. 13:**
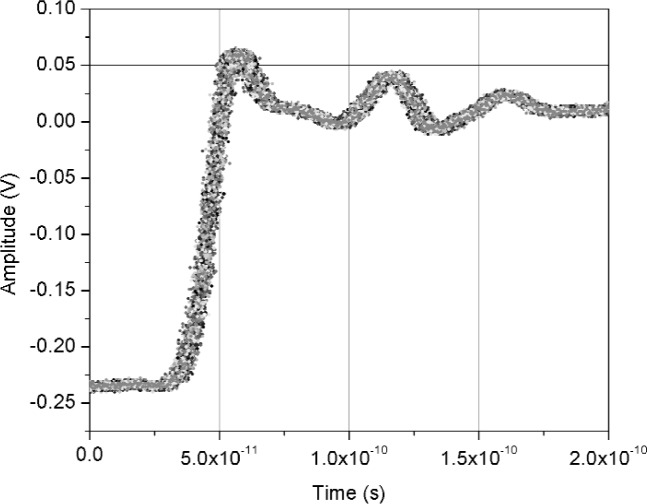
Set of jittered noisy waveforms acquired from sampler A, where the input jitter value was measured to be 1.45 ps rms.

**Fig. 14:**
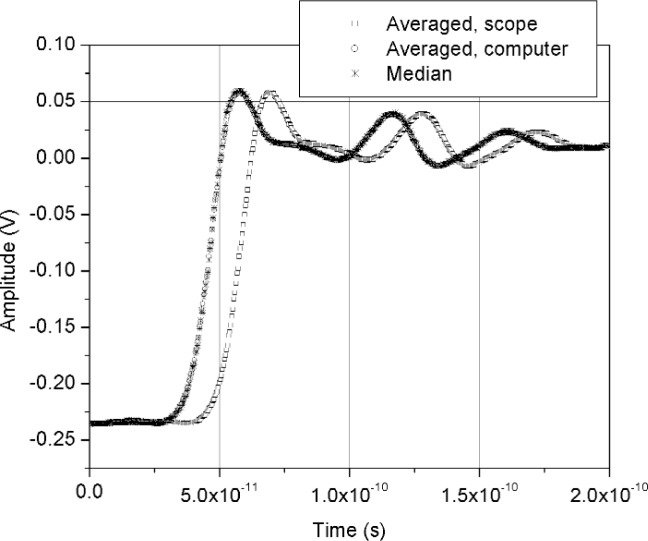
The scope averaged, computer averaged, and median reconstructed waveforms corresponding to the data set shown in Fig. 13. The delay between waveforms is an artifact of the acquisition process and has no effect on the analyses presented here.

**Fig. 15:**
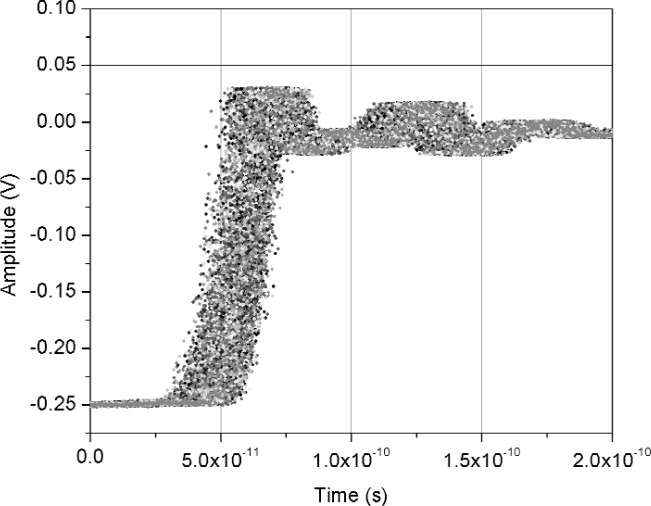
Set of jittered noisy waveforms acquired from sampler B, where the input jitter value was measured to be 6.44 ps rms.

**Fig. 16:**
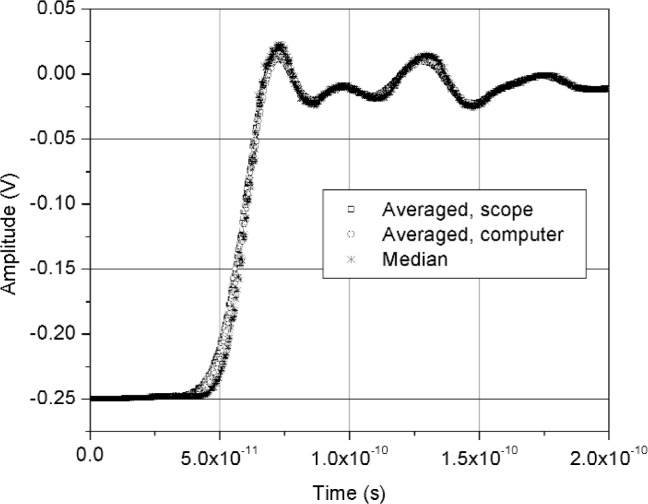
The scope averaged, computer averaged, and median reconstructed waveforms corresponding to the data set shown in Fig. 15.

**Fig. 17:**
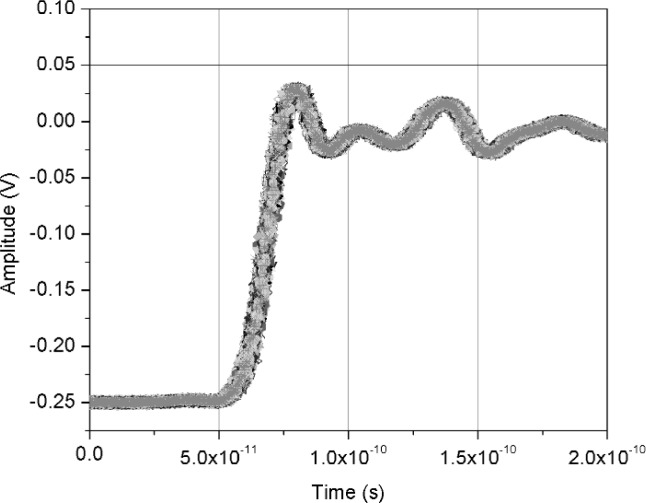
Set of jittered noisy waveforms acquired from sampler B, where the input jitter value was measured to be 0.92 ps rms.

**Fig. 18:**
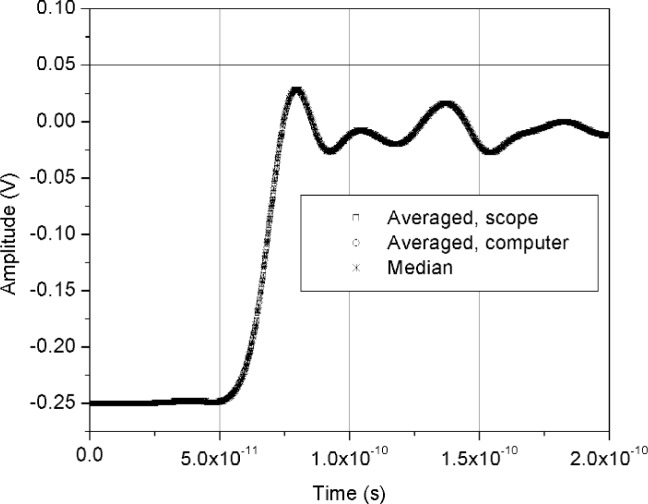
The scope averaged, computer averaged, and median reconstructed waveforms corresponding to the data set shown in Fig. 17.

Figures 11 to 18 show the results of experiments using two different sampling oscilloscopes (samplers), sampler A and sampler B, one each from a different manufacturer with different levels of jitter. The jitter was varied by adjusting the trigger level on the oscilloscope. All trigger jitter values were obtained using the oscilloscope firmware. The 3 dB attenuation bandwidths for both samplers are nominally 50 GHz. The pulses produced by the pulse generator have a nominal transition duration of 12 ps. The sampling intervals were 2 ps. There were *M* = 128 waveforms in each set of waveforms.

Figure 11 shows the results of 128 waveforms acquired using sampler A with a jitter level of 9.5 ps rms. The transition duration of the cumulative jitter distribution is about 24.3 ps. Figure 12 shows the oscilloscope-averaged waveform, the mean computed from each of the waveforms shown in Fig. 11, and the waveform reconstructed using the median method. Table 1 shows the transition duration values computed for these three cases. Figures 13 and 14 show the same results as Figs. 11 and 12 except for an rms jitter value of 1.5 ps. The transition duration of the oscilloscope-averaged waveform was not equal to that obtained from the computer-averaged waveform. This may in part be explained by the fact that the waveforms represented by the oscilloscope-averaged waveform were not the same as those used for the computer-averaged waveform. This discrepancy may not have been as large if the number (128) of waveforms to generate the averaged waveforms shown in Figs. 11–14 was larger. Figures 9 and 10 show that around 500 waveforms should be averaged to converge to a consistent value. However, for the low jitter case, the differences between the transition durations of the computer- and scope-averaged waveforms are within the uncertainties of the measurement [3]. The differences in the transition durations of the median-reconstructed waveforms for both levels of jitter are within measurement uncertainty.

Figures 15 through 18 and Table 2 show the same results for Sampler B as Figs. 11 through 14 and Table 1 did for Sampler A, except with different levels of trigger jitter. The same observation made for Sampler A and the low jitter case is the same for Sampler B (see Tables 1 and 2). For the large jitter case for Sampler B, however, the transition duration of oscilloscope-averaged waveforms was greater than that of the computer-averaged waveforms. Also, the transition durations of the median-reconstructed waveforms were faster for Sampler A than for Sampler B, indicating that the bandwidth of Sampler A is greater than that of Sampler B. This is corroborated by the low jitter results (see Tables 1 and 2 first row) where it can be approximated that the contribution of jitter to *t*_sys_ is negligible and, given the same pulse source, the difference in transition duration is caused by the difference in *t*_instr_ of the samplers.

Table 3 shows the results of limiting the number, *M*, of waveforms used to compute the median on the value of transition duration. This is analogous to the simulation results shown in Fig. 10 except that only one set of *M* waveforms is used in Table 3 whereas 100 sets of *M* waveforms were used to produce the results shown in Fig. 10. Table 3, however, still shows the importance of selecting an appropriate value of *M*, namely one that is adequate to obtain reproducible results.

## 5. Aberration Amplitudes

As mentioned earlier, a waveform reconstructed using the median method will exhibit an underestimate of the magnitude of aberrations. Typically, however, it is difficult to estimate the uncertainty in overshoot (*OS*) and undershoot (*US*) (both terms are defined in Ref. [6]) of the reconstructed waveform because *OS* and *US* in this waveform are affected by a variety of input signal and measurement instrument characteristics [3]. Consequently, we developed an empirical method to estimate the uncertainty in *OS* and *US* of the median reconstructed waveforms using the *OS* and *US* values from the acquired waveform. This uncertainty is estimated by the difference, *d*_max_, between the jitter-caused attenuation of a sinewave and that of a constant signal. The sinewave has a half-period equal to the duration, *T*_abb_, of the *OS* or *US* from the acquired waveform. *d*_max_ is given by:

$$\begin{array}{c} d_{\max}=\frac{V_{\text{abb}}\int_{t=0}^{kT_{\text{PD}}}(1-\cos\;[2\pi \;f_{\text{abb}}t])dt}{\int_{t=0}^{kT_{\text{PD}}}dt}\\ =V_{\text{abb}}\frac{\left[kT_{\text{PD}}-\frac{\sin(2\pi \;f_{\text{abb}}kT_{\text{PD}}}{2\pi \;f_{\text{abb}}}\right]}{kT_{\text{PD}}}\\ =V_{\text{abb}}\;\left[1-\frac{\sin\;(2\pi \;f_{\text{abb}}kT_{\text{PD}})}{2\pi \;f_{\text{abb}}kT_{\text{PD}}}\right], \end{array}$$ (6)

where *T*_PD_ is the width of the probability density function of the jitter, *V*_abb_ is the amplitude of the aberration, *k* is an empirically-determined factor adjusted to ensure *d*_max_ is greater than the measured error (in situations where it is possible to measure the error), *f*_abb_ = 0.5/*T*_abb_, and *T*_abb_ is the duration of the aberration. As a check for Eq. (6), if *T*_PD_ = 0 (no jitter), then *d*_max_ = 0 (no uncertainty contribution from the jitter), as would be expected. On the other hand, if *T*_PD_ = *N*/*T*_abb_, where *N* is an integer, then *d*_max_ would approach *V*_abb_ as *N* increased. This also makes sense because as *N* increases, jitter would tend to cause the aberration to disappear in the observed waveform. The expanded uncertainties for *OS* and *US* should include *d*_max_ as well as contributions from other sources [3]. Simulations were performed that yielded 25 sets of data for four different levels of noise and different values of *T*_abb_. Each set of data was the result of the average of 256 waveforms. As shown in Table 4, the results from the analysis of this simulated data show that Eq. (6) provides an overestimate of the errors in *OS* values. With respect to fidelity of reconstruction of the aberrations in the waveform, “monotonicity” is satisfied if *σ*_jit_ ≤ 0.2 *T*_abb_. The value of *k* = 4 was used in Table 4 to ensure that uncertainty in *OS* for the noisier signals would be less than *d*_max_. This method for finding a limit in the *OS* or *US* uncertainty when *σ*_jit_/*T*_abb_ = 2 did not work because this amount of jitter effectively removed the aberration from the observed waveform.

**Fig. 19:**
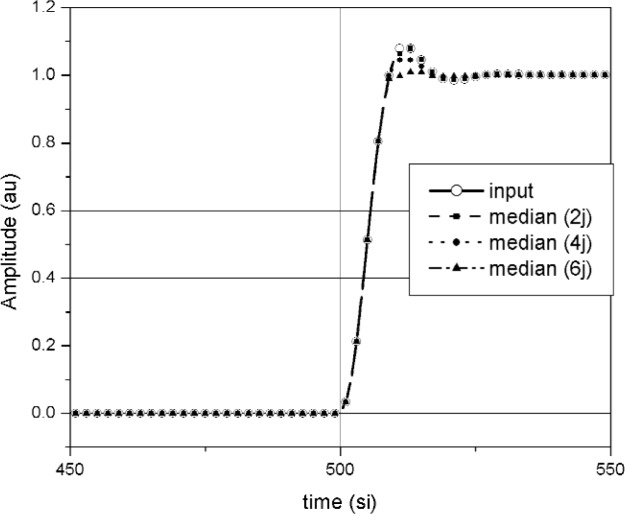
The input waveform and the waveforms reconstructed using the median method for different values of jitter as indicated by “2j,” “4j,” and “6j.” The jitter is in units of si; therefore, 2j corresponds to jitter of 2 si.

**Fig. 20:**
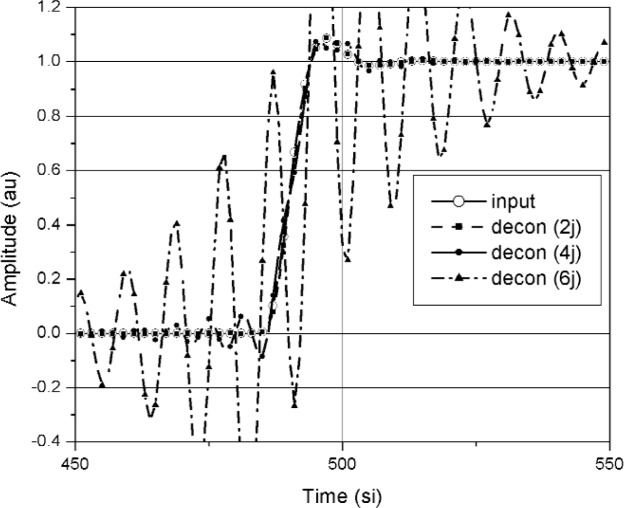
The input waveform and the waveforms reconstructed using the jitter deconvolution method for different values of jitter as indicated by “2j,” “4j,” and “6j.” The jitter is in units of si; therefore, 2j corresponds to jitter of 2 si.

**Fig. 21:**
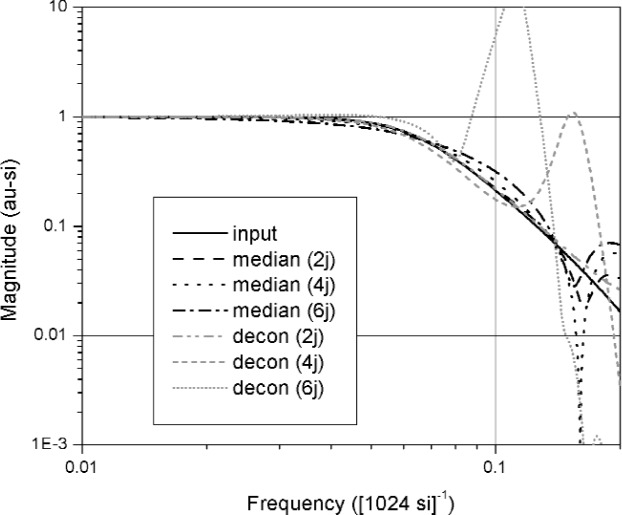
The spectra of the input waveform and the waveforms reconstructed using the median and jitter deconvolution methods for different values of jitter as indicated by “2j,” “4j,” and “6j.” The jitter is in units of si; therefore, 2j corresponds to jitter of 2 si.

A comparison of waveforms reconstructed using the median method and jitter deconvolution are shown in Figs. 19–21. Figures 19 and 20 show waveforms reconstructed using the median and jitter deconvolution methods respectively. These figures show that for large jitter, the median method provides a much more realistic reconstruction of the input signal than does the jitter deconvolution method. This is corroborated by the distorted spectra caused by the jitter deconvolution method (see Fig. 21). For small levels of jitter, *t*_jit_ ≤ *t*_instr_, the jitter deconvolution and median methods appear to work equally well for removal of jitter from the waveforms.

## 6. Discussion

The simulation results of Secs. 2 and 3 and experimental results of Sec. 4 can be compared by multiplying the simulation horizontal axis values and corresponding waveform parameters by two and then changing the simulation horizontal axis units from si to ps. Accordingly, the jitter of 2 si becomes 4 ps and the simulated transition duration of 5.86 si becomes 11.72 ps, which corresponds to the transition duration of typical high-speed pulse generators. The simulated trigger jitter of 4 ps is at least three times larger than what is typically observed. Consequently, the difficulty in reconstructing the aberrations of a waveform that was experienced in the simulation would not be experienced in reconstructing actual signals used in waveform metrology. In general, the simulation and measurement results show that the median method for reducing the effects of trigger jitter and noise on the acquired waveform is effective. However, to accurately reproduce waveform aberrations, the duration of the aberrations should not be greater than the transition duration of the cumulative jitter distribution.

## Figures and Tables

**Table 1:** Transition duration values computed for waveforms taken from Sampler A

Jitter (ps)	Averaged, scope (ps)	Averaged, computer (ps)	Median (ps)
1.5	12.9	12.7	12.2
9.5	23.1	26.3	12.6

**Table 2:** Transition duration values computed for waveforms taken from Sampler B

Jitter (ps)	Averaged, scope (ps)	Averaged, computer (ps)	Median (ps)
0.9	13.3	13.4	13.3
4.7	16.8	15.6	13.2

**Table 3:** Transition duration values for waveforms acquired using Samplers A and B. *t*_meas_ is the measured transition duration

Sampler (*σ*_jit_)	A (1.5 ps) *t*_meas_ (ps)	A (9.5 ps) *t*_meas_ (ps)	B (0.9 ps) *t*_meas_ (ps)	B (4.7 ps) *t*_meas_ (ps)
*M*				
2	11.4	10.7	11.5	10.3
4	12.2	19.1	13.0	9.7
8	12.5	6.4	13.1	8.4
16	11.8	9.5	13.1	11.2
32	12.4	9.3	13.4	11.3
64	12.5	9.4	13.3	13.0
128	12.2	12.6	13.3	13.2

**Table 4:** The effect of *σ*_jit_ on *OS* for a given *T*_abb_ with and without noise. The four rows in each cell of the table are the *OS* value obtained from the simulation, *d*_max_; the estimated value, *OS*_sm_, plus *d*_max_; and the standard deviation of the observed values, *σ*_OS_. For the data in this table, *k* = 4 and *T*_abb_ = 5.293 si. The bottom row shows the value of *OS* for the noise-free, jitter-free input signal

*σ*_jit_/*T*_abb_		0.1	0.2	*OS* (au) 0.5	1.0	2.0
*σ_n_* (au)						
0	*OS*_sm_	0.05747	0.05747	0.05747	0.01472	0.00495
	*d*_max_	0.00530	0.02057	0.02057	0.06558	0.00001
	*OS*_sm_+*d*_max_	0.06277	0.07804	0.07804	0.08030	0.00496
	*σ*_OS_	± 2×10^−13^	± 2×10^−12^	± 2×10^−12^	± 1.3×10^−2^	± 1.7×10^−7^
0.001	*OS*_sm_	0.05748	0.05744	0.05744	0.01472	0.00495
	*d*_max_	0.00524	0.01992	0.01992	0.06558	0.00001
	*OS*_sm_+*d*_max_	0.06272	0.07736	0.07736	0.08030	0.00496
	*σ*_OS_	± 2.7×10^−5^	± 1.610^−4^	± 1.7×10^−4^	± 2.2×10^−2^	± 2.4×10^−7^
0.01	*OS*_sm_	0.05755	0.04688	0.04688	0.01634	0.00498
	*d*_max_	0.00505	0.01697	0.01647	0.05896	0.00930
	*OS*_sm_+*d*_max_	0.06260	0.06385	0.06335	0.07530	0.01428
	*σ*_OS_	± 2×10^−4^	± 7.410^−4^	± 7.4×10^−4^	± 1.6×10^−2^	± 1×10^−2^
0.1	*OS*_sm_	0.05576	0.05055	0.05055	0.0367	0.02595
	*d*_max_	0.00516	0.02262	0.02262	0.1351	0.00280
	*OS*_sm_+*d*_max_	0.06082	0.07317	0.07317	0.1718	0.02575
	*σ*_OS_	± 9×10^−3^	± 2×10^−3^	± 2×10^−2^	± 1.2×10^−2^	± 7×10^−3^

Input *OS* (au)		0.05474	0.05474	0.05474	0.05474	0.05474

## References

[1] Abramowitz M, Stegun IA (1964). Handbook of Mathematical Functions with Formulas, Graphs, and Mathematical Tables.

[2] Gans WL (1983). The measurement and deconvolution of time jitter in equivalent-time waveform samplers. IEEE Trans Instrum Meas.

[3] Paulter NG, Larson DR (2002). Pulse parameter uncertainty analysis. Metrologia.

[4] Nahman NS, Guillaume ME (1981). Deconvolution of time domain waveforms in the presence of noise.

[5] Paulter NG (1994). A causal regularizing deconvolution filter for optimal waveform reconstruction. IEEE Trans Instrum Meas.

[6] (2003). IEEE Standards on Transitions, Pulses, and Related Waveforms, Std 181-2003.

[7] Souders TM, Flach DR, Hagwood C, Yang GL (1990). The effect of timing jitter in sampling systems. IEEE Trans Instrum Meas.

[8] Verspecht J (1994). Compensation of timing jitter-induced distortion of sampled waveforms. IEEE Trans Instrum Meas.

[9] Cox MG, Harris PM, Humphreys DA (1993). An algorithm for removal of noise and jitter in signals and its application to picosecond electrical measurement. Numerical Algorithms.

